# Superb microvascular imaging (SMI) and elastosonography in thyroid nodule: diagnostic value in a real-time cohort

**DOI:** 10.1007/s40477-024-00898-5

**Published:** 2024-07-05

**Authors:** Davide Negroni, Gaetano Maddalena, Romina Bono, Flavia Abruzzese, Sara Cesano, Patrizio Conte, Chiara Airoldi, Pierluigi Neri, Alessandro Carriero

**Affiliations:** 1 Department of Radiology, “Maggiore della Carità” Hospital, Novara, Piedmont Italy; 2 Department of Translation Medicine, University of “Piemonte Orientale”, Novara, Piedmont Italy

**Keywords:** Superb microvascular imaging, SMI, Elastonography, TI-RADS, Thyroids nodule, Diagnostic power

## Abstract

**Purpose:**

In clinical practice, thyroid nodules are classified according to TI-RADS by B-mode and color-flow Doppler study. The aim of the study is to evaluate the possible added value of Superb microvascular imaging (SMI) and elastosonography in the stratification of malignancy risk of thyroid nodules.

**Methods:**

All patients with thyroid nodules who were candidates for needle aspiration were enrolled. Experienced operators performed a standard examination with TI-RADS calculation, followed by SMI and elastosonography on the nodules. The needle aspiration outcome was used as the gold standard. Statistical analysis calculated the ROC curves of the techniques applied individually and serially.

**Results:**

In this prospective study, we analysed 260 nodules, found in 251 patients (mean age 58.6 yo ± 14). 11.2% were TI-RADS 1, 18.9% TI-RADS 2, 41.1% TI-RADS 3, 28.1% TI-RADS 4, and 0.8% TI-RADS 5.

The SMI technique showed an AUC of 0.57 (95% CI 0.49; 0.66) while elastosonography had an AUC of 0.58 (95% CI 0.49; 0.67) when used individually. SMI together with elastosonography had AUC of 0.62 (95% CI 0.52; 0.71). TI-RADS had AUC of 0.67 (95% CI 0.59; 0.75). SMI and elastosonography applied together with TI-RADS had AUC of 0.69 (95% CI 0.61; 0.77).

**Conclusion:**

In the real-world cohort of patients, the SMI technique and elastosonography slightly increase the AUC of TI-RADS. Taken individually, SMI and elastosonography do not have a very strong AUC.

## Introduction

Thyroid nodules are common and can be found in 33% of the adult population between the ages of 18 and 65 years and in 50% of adults over 65 years old [1–3]. Fortunately, only a small percentage of nodules is malignant (prevalence of 5–15%) and thyroid cancer has a slow progression and a very good prognosis [3–5].

Computed tomography (CT) scan and Magnetic Resonance (MRI) are not useful in the detection and evaluation of thyroid nodules [3, 4, 6]; Ultrasound is more accurate and useful but is not considered sufficiently sensitive and specific in the differentiation between benign and malignant nodules.

Thyroid Imaging Report and Data System (TI-RADS) considers these parameters as suspicious of malignancy: hypoechogenicity, microcalcifications, irregular margins, solid composition, and a taller-than-wider shape. Unfortunately, the US can detect suspicious nodule characteristics but not all of these are always present in malignant nodules, and some are found also in benign ones.

In addition to the standard B-mode evaluation also the colorDoppler one is usually performed. This technique allows for evaluating the nodule’s vascularization that can present different vascular patterns: no vascularity, only perinodular vascularity, and mild or moderate intranodular vascularity with or without the perinodular one [6, 7]. TI-RADS do not include lymph nodes or elasticity in the evaluation [8]. Only peripheral vascularity is suggestive of benignity whereas the intranodular one can be present both in malignant and benign nodules For this reason the role of this type of evaluation is uncertain and vascular pattern can not be used to have a diagnosis of malignancy.

In our study, we mainly focused on the role of elastography and Superb Microvascular Imaging (SMI) in thyroid nodule evaluation.

Superb Microvascular Imaging (SMI) is a technology able to detect very small vessels in the nodule. Different studies showed that SMI is more useful in vasculature characterization compared to colorDoppler and Power Doppler techniques in the detection of penetrating vessels [9–11]. Chen and colleagues also showed that the addition of SMI evaluation could increase the performance of TIRADS.

Elastosonography is a US technique that can estimate the tissues' elasticity. Currently, two different techniques exist, which are the “Strain” technique which is based on manual compression, and the “Shear Wave” technique which shows a map indicative of the local elasticity of the tissues in real-time without any compression of the organ [12–14].

It is based on the application of an external force (manual compression with the probe) that induces tissue deformation that is greater in soft tissues and lesser in hard ones. This technique can be applied to the evaluation not only of thyroid nodules but also of breast, lymph nodes, and musculoskeletal system [15]. This is a painless examination that can be easily performed during a normal US examination. Different studies showed that thyroid cancer is associated with greater stiffness whereas benign lesions are usually softer [12, 16–18].

Different studies showed that elastography could be a good alternative to fine needle aspiration cytology in the differential diagnosis of thyroid nodules. Of course, this technique has its limits, particularly in the diagnosis of non-stiff thyroid tumors such as follicular carcinomas that can be less stiff like benign lesions [6, 12]. Also, fibrosis could lead to a wrong interpretation since it may be present on both benign and malignant nodules [4]. Of course, another limitation is that Strain Elastography is examiner-dependent and requires an experienced operator.

Fine needle aspiration cytology is considered the gold standard in the diagnosis of thyroid cancer. The main diagnostic problem is the category of indeterminate cytology which has a mean cancer risk of 16%. A study showed that including elastography in the TI-RADS could increase its diagnostic performance in these indeterminate cases and could decrease the number of diagnostic surgeries [12].

This study aims to describe the diagnostic power of TI-RADS, elastosonography, and SMI in a real-world cohort and the possible added value.

## Material and methods

Between January 2021 and July 2022 253 patients for a total of 265 nodules scheduled to undergo ultrasound-guided FNA, were enrolled in this prospective single-center study.

This sample represents a consecutive court of patients referred by different medical specialists presenting at least one thyroid nodule and candidate for FNA. The criteria for applying to the FNA were heterogeneous (features the US, risk factors, blood chemistry tests, etc.).

No exclusion criteria were applied in the study, except for not signing the consent to the data collection.

Informed consent for ultrasound elastography and SMI was obtained from patients.

### Ultrasonography examination

The patients had already carried out the B-mode and ECD examination at another site where the indication for the execution of the FNAC was indicated.

Four radiologists (one with more than twenty years of experience and three postgraduates with six months of experience in thyroid ultrasound) acquired B-mode, ECPD, SMI, ES Strain images before the FNAC.

The hardware used was Aplio 500 (Toshiba Medical Systems Corp., Tokyo, Japan) with a linear probe PLT-1005BT 10–14 MHZ for the elastosonography.

The B-mode was completed by colorDoppler and PowerDoppler, collecting explanatory 2dDimaging.

**Fig. 1:**
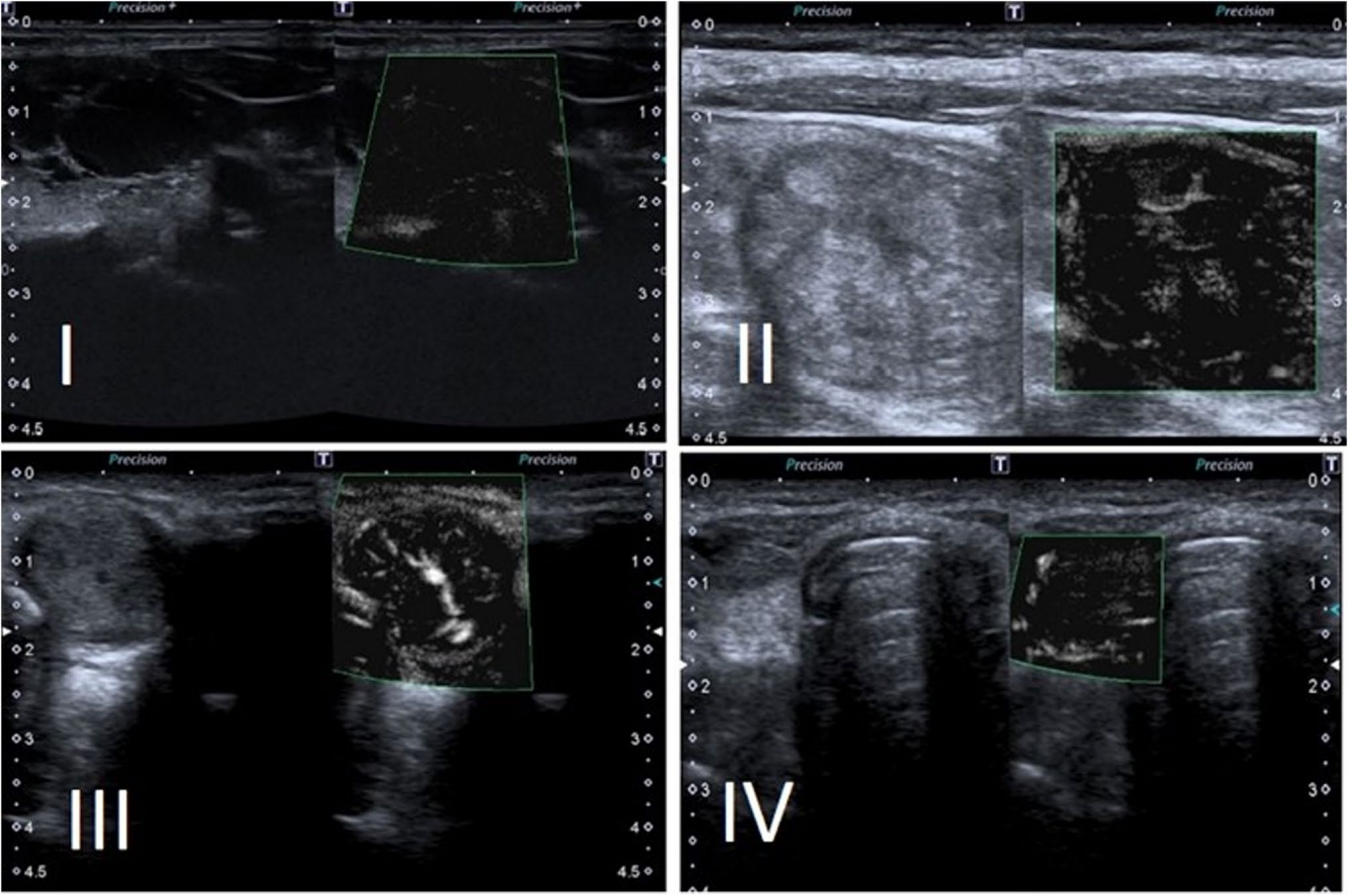
Schematic of classification based on the distribution patterns of nodule vascularity on superb microvascular imaging. Type I: absence of nodule vascularity. Type II: predominantly perinodular vascularity with continuous (IIa) or discontinuous circumferential (IIb) vascularity at the margin of a nodule. Type III: predominantly intranodular vascularity, linear (IIIa), branching (IIIb) or diffusing (IIIc) with or without perinodular vascularity. Type IV: penetrating vascularity with (IVa) or without (IVb) perinodular vascularity of a nodule

Without changing the patient's position, and on the same B-mode slice, the two Radiologists.

acquired the SMI imaging. The SMI examination was performed in monochromatic mode, with a focus at the target nodule height with scale 3.4 58 fps, G:85, DR: 65, CG:42, 19.8 k, F:0; a ROI rectangular-shaped was focused on the target lesion and adjusted to include surrounding normal thyroid and adjacent muscle (pre-thyroids muscle, sternocleidomastoid muscle), in according to Ahn et al. [19] (Fig. 1). At least three 2D imaging were acquired during the samples e three clips about PowerDoppler, colorDoppler, SMI.

**Fig. 2:**
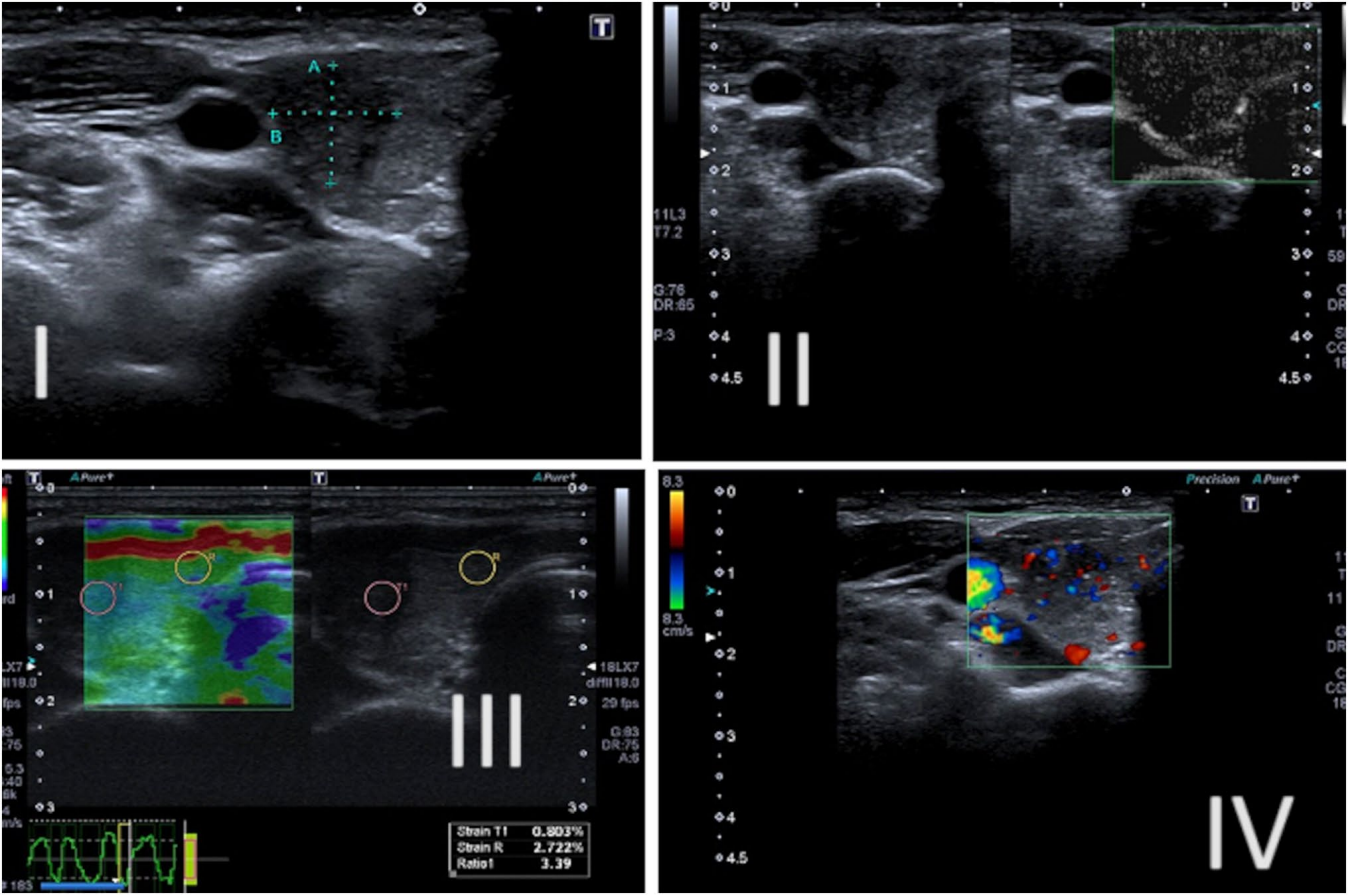
Example patient with a nodule classified as TI-RADS 4 (hypoechogenic, with well-defined margins and no calcifications). (I) B mode; (II) Superb Microvascular Imaging (SMI); (III) Elastosonography with diagnostic correctness graph; (IV) colorDoppler. The SMI study shows type IV vascularisation. Elastonosonography reports a Strain Ratio of 3.39. The nodule was malignant (Thy 5) on needle aspiration

The strain elastography was conducted using repeated vertical manual compression; the software available scheduled a color box to adjust the speed and pressure and standardize the activity (Fig. 2); all the operators followed the software's correctness criteria for the elastosonography and analyzed a clip, evaluating as a frame, the decompression zone of the nodule.

When the reference area appeared as a mix of red and green color, at least 2 representative 2D imaging were acquired.

Ultrasound-guided FNA was conducted and analyzed by a team of expert pathologists (more the 10 years of experience in thyroid FNA) using a 23-gauge needle attached to a 5 ml syringe.

Through the nodule, several multidirectional FNAs were performed to increment the sampling success [3, 20]. Specimens were preserved in bottles with 95% ethanol for liquid-based cytological examination. Cytopathological reports of FNA were prepared according to the UK Royal College of Pathologists thyroid fine-needle aspiration [20].

No biopsies were conducted on the target nodule.

### Data and statistical analysis

All the collected images were analyzed anonymously and independently by two other expert Radiologists (with about 10 years of experience in thyroid imaging), blinded to the FNA result; the two Radiologists expressed unanimously a report.

From the B-mode examination data about composition, echogenicity, shape, margin, and echogenicity foci were collected, according to the American College of Radiology criteria. The ACR TI-RADS value was calculated again, to express the degree of probability of clinically detectable disease:

- TR1: 0 points
  - benign
- TR2: 2 points
  - not suspicious
- TR3: 3 points
  - mildly suspicious
- TR4: 4–6 points
  - moderately suspicious
- TR5: ≥ 7 points
  - highly suspicious

The powerDoppler and colorDoppler signals were classified as (1) none, (2) predominant perivascular, (3) predominant intravascular, (4) mixed [7, 21].

The SMI signal was reported according to and citing Shin et al. as 4 type: “type 1, absence of nodule vascularity; type 2, predominant intranodular with continous or discontinous perif vascularity only (presence of circumferential vascularity at the margin of a nodule); type 3, mild intranodular vascularity with or without perinodular vascularity (vascularity lesser than 50%); type 4, marked intranodular vascularity with or without perinodular vascularity (vascularity greater than 50%)” [22].

Color-coding of elastographic images was conducted according to the scoring system recommended by Asteria et al., and red/green and blue represented soft and hard tissues, respectively [23].

The FNA results were classified following the Guidance on the reporting of thyroid cytology specimens (January 2016) of the Royal College of Pathologists [20] as:

- Thy 1/Thy 1c: Non-diagnostic for cytological diagnosis
- Thy 2/Thy 2c: Non-neoplastic
- Thy3: Neoplasm possible
- Thy4: Suspicious of malignancy
- Thy5: malignant

## Results

A total of 253 patients (70.3% male, 29.7% female) were enrolled in the study. Five subjects refused to give consent to perform the examination (Table 1).

**Table 1:** The table summarises the principal findings of the examined sample

	*N*	%
M	183/260	70,40%
F	77/260	29,60%
Mean age	58.6 ± 12.3	
Site		
Left	108/260	41.50%
Right	129/260	49.60%
Istmo	23/260	8.90%
Subcapsular	0/260	
Mean Dmax	19.6 ± 9.6	
Mean volume	6402.7 ± 8107.5	
TI-RADS		
1	29/260	11.10%
2	50/260	19.20%
3	107/260	41.20%
4	73/260	28.06%
5	1/260	0.04%
ASTERIA		
1	42/260	16.20%
2	215/260	82.70%
3	3/260	1.10%
4	0/260	0%
Mean strain ratio	1.69 ± 1.3	
SMI		
1	11/260	4.20%
2	85/260	32.70%
3	126/260	48.50%
4	38/260	14.60%
THY FNA		
1	35/260	13.50%
2	173/260	66.50%
3	36/260	13.80%
4	6/260	2.30%
5	10/260	3.90%

*M* male, *F* female, *Dmax* max dimension, *SMI* superb microascular imaging, *FNA* fine needle aspiration, *ASTERIA* ASTERIA criteria

The mean age of the patients was 58.6 years with a standard deviation of 12.3 years.

The SMI technique and the technique of elastosonography were successfully performed in 100% of the subjects. The average time for image acquisition of the individual techniques was 2 min ± 1 for SMI and 4 min ± 1 for elastosonography.

The characteristics of the nodules analyzed were represented schematically in Table 1.

All the identified nodules underwent FNAC; the examination reported a success rate in 86.5% of the sampling (13.5% of the FNAC were inadequate with Thy = 1).

Of the 260 nodules submitted to FNAC, 16 reported a Thy > 3 (6.2%). The percentage of nodules classified as “non-neoplastic” on the FNAC examination was 173/260 (66.5%). In 36/260 (13.8%) nodules the Pathologist reported a Thy = 3.

According to TI-RADS classification, the most present pattern on B-mode examination was TI-RADS 3, present in 107/260 (41.2%) nodules, followed by TI-RADS 4 present in 73/260 (28.1%) nodules, and TI-RADS 5 in 1/260 (0.04%). “Benign” TI-RADS, below grade 3, accounted for 30% of the nodules analyzed.

The ROC curve constructed on TI-RADS values reported an Area Under the Curve (AUC) of 0.67 (95% CI 0.60; 0.75). Statistical analysis showed that the variables TI-RADS and malignancy at FNAC were dependent (*p* < 0.05) The Odd Ratio (OR) for TI-RADS 3 was 3, for TI-RADS 4 was 5.6, and for TI-RADS 5 was 13.46. The Likelihood Ratio was largely increased, of 17.1.

PowerDoppler and colorDoppler presented type IIIb as the most frequent pattern in 62.4% of patients, followed by type 2 in 33.0% and type 1 in 4.6%. Statistical analysis did not observe a correlation between the type of vascularisation and the nature of the nodule (*p* = 0.45).

The SMI reported the type 3 pattern, “mild intranodular vascularity with or without perinodular vascularity (vascularity lesser than 50%),” in the majority of 126/260 (48.5%) nodules, followed by type 2, “predominant intranodular with continuous or discontinuous peripherical vascularity only (presence of circumferential vascularity at the margin of a nodule)” present in 85/260 (32.7%) of nodules.

The ROC curve constructed on the data collected by the SMI technique showed an AUC of 0.57 (95% CI 0.49; 0.66). SMI e malignità alla FNAC non sono risultate essere dipendenti (*p* > 0.05). The OR for SMI type 2 was 1.12, for SMI type 3 was 1.51, and for SMI type 4 was 2.73. No nodule was classified as SMI type 1.

Elastosonography presented a statistically significant difference (*p* < 0.05) between benign and malignant nodules. The mean Strain Ratio in nodules with malignancy on cytological examination was 2.20 with a standard deviation of 1.23, while in benign nodules on cytological examination it was 1.5 with a standard deviation of 1.04.

The elastography ROC curve reported an AUC of 0.56 (95% CI 0.48; 0.67). Statistical test was not significant (*p* > 0.05). The OR for a Strain Ratio of 2.20 was 1.18.1

Using together and exclusively (without TI-RADS calculation), elastosonography and SMI presented an AUC at the ROC curve of 0.62 (95% CI 0.52; 0.71). The variable “elastosonography + SMI” was not found to be dependent on malignancy at FNAC (*p* > 0.05). The Likelihood Ratio chi 2 (LR) is moderately increased, of 6.02.

Combining the two variables SMI and elastosonography with TI-RADS observed an AUC of 0.69 (95% CI 0.60; 0.76) (SMI + TI-RADS), and 0.70 (95% CI 0.62; 0.77) (elastosonography + TI-RADS), respectively. An increase in LR of 18.5 and 19.5 was observed in both cases, respectively.

Analysis by *t* test was not significant for volume and malignancy comparison at FNAC (*p* > 0.05).

## Discussion

Elastosonography and Superb Microvascular Imaging (SMI) describe different aspects of a nodule. Tissue elasticity and microvascularisation could better typify the thyroid nodule. This study aims to analyze the possible additional diagnostic ability of the SMI technique and elastosonography in the diagnosis of thyroid nodules.

The sample was drawn from normal room activity practice, involving all patients who required needle aspiration of the nodule. Given the absence of exclusion criteria and biased selection, it was considered a real-world study.

In our sample, the percentage of malignancy found at FNAC was lower than reported in the literature. The prevalence of malignancy in cohorts of patients with thyroid nodules is about 10% [3, 8, 12]. In the TI-RADS classification, points are given according to the characteristics of the nodules, and the risk classification of the nodules is made considering the total score [8]

The most represented category of TI-RADS was the value of TI-RADS 3 (44%).

While the prevalence rates of TI-RADS 4 and TI-RADS 5 are similar to those described in the literature [2, 12, 24, 25], in this study the percentage of TI-RADS 2 and 3 disagree. The high number of these categories may be due to the nature of the study: being a real-world cohort, many nodules were sent with no apparent evidence of malignancy or only for further diagnosis.

TI-RADS was found to be the variable most strongly associated with nodule malignancy. The score performance was inferior to that reported in the meta-analysis by Provenzale et al.: the author estimated an AUC of 0.86 (95% CI 0.83; 0.89) for ACR TI-RADS [26].

The difference in diagnostic performance may be due to the limited sample of the study.

The highest incidence of malignancy was found in ACR TI-RADS 4 and 5. FNAC is mandated in these classes [25, 27]. Attention should also be paid to TI-RADS 3 where 3 malignant nodules were found. Large population studies showed the incidence of malignancy in TI-RADS 3 was estimated to be 4.8% [2, 8, 28]. In this category, follow-up is therefore recommended [8].

The study by elastosonography, when used alone, was not able to discriminate between benign and malignant nodules. An increased stiffness of the nodule, however, was found in all cases of malignancy [14, 17, 23]. The finding in the literature is still debated.

Asteria et al., in a study on benign and malignant thyroid nodules, described a high predictive ability of elastosonography in the diagnosis of malignancy, about 94% [23]. Colorimetric scales based on elastosonography appear to have a high number of false positives [29].

The difference between the data found in our study and the study by Asteria et al. may be due to the higher benign-to-malignant ratio in our cohort.

The result of colorDoppler and powerDoppler confirms what Moon et al. and Tamsel: the description of the vascularisation alone or in combination with the B-mode was not useful for predicting thyroid malignancy [7, 21]. Indeed, the benign thyroid nodules may also present a marked intranodular (type III pattern) blood flow. Due to the limited number of TI-RADS 4 and 5, no stratified analysis was performed for this category.

Analyzing the ROC curves, the addition of elastosonography to the TI-RADS calculation slightly increases both AUC and LR.

Given the data found in this study and what has been reported in the literature, elastosonography appears to have added value over B-mode. However, its performance must be followed sequentially to the TI-RADS calculation to exclude nodules with a liquid appearance. A limitation to the interpretation of the data may be the distance from the carotid artery. Kim et al. noted that the accuracy of elastosonography depends on the distance from the carotid artery [16]. This was not considered in the study. Another limitation is the absence of a pathological cohort. Follicular carcinomas may present similar rigidity to benign nodules [12].

The time taken to acquire images with elastosonography was shorter than documented by Asteria et al. [23]. The difference could be due to the presence of only one experienced radiologist during the examination and not two operators.

As far as the SMI was concerned, the reasoning is the same. In the literature, some authors have observed an improvement in diagnostics using the SMI. Many studies found an SMI sensitivity value between 76 and 78% and specificity values between 93.6 and 95% [10, 11, 29]. Moreover, the Korean Society of Thyroid Radiology supports the use of SMI as a discriminatory technique between benign and malignant nodules [6].

However, the finding was only partially confirmed in our real-world cohort. The SMI slightly increased the AUC of TI-RADS, as does the LR, but not significantly. The value, therefore, deviates from what has been reported in the literature, however, our cohort includes nodules from different TI-RADS, especially TI-RADS 3.

Once the TI-RADS has been calculated, the use of the two techniques is recommended to strengthen the malignancy hypothesis.

Future studies may aim to describe the anatomopathological appearance of thyroid glands and their surgically removed nodules. In addition, the size of the cohort studied will be expanded.

## Conclusions

In the real-world cohort of patients analyzed in this study, the TI-RADS, elastosonography, and SMI showed lower diagnostic performance than expected TI-RADS. Performing elastosonography and SMI after TI-RADS can help identify the nature of the nodule. Solid nodules appear at greater risk of malignancy. In the study of vascularisation with SMI, no specific patterns of evolving lesions were identified. Further large population studies are needed.
