# Correction to: Superb microvascular imaging (SMI) and elastosonography in thyroid nodule: diagnostic value in a real‑time cohort

**DOI:** 10.1007/s40477-024-00978-6

**Published:** 2025-01-13

**Authors:** Davide Negroni, Gaetano Maddalena, Romina Bono, Flavia Abruzzese, Sara Cesano, Patrizio Conte, Chiara Airoldi, Pierluigi Neri, Alessandro Carriero

**Affiliations:** 1 Department of Radiology, “Maggiore della Carità” Hospital, Novara, Piedmont Italy; 2 Department of Translation Medicine, University of “Piemonte Orientale”, Novara, Piedmont Italy

**Correction to: Journal of Ultrasound** 10.1007/s40477-024-00898-5

The author name Dr. Pierluigi Neri has been missed out in the original publication by mistake. He has been included in the author group. The complete correct order of author should read as follows.

Davide Negroni, Gaetano Maddalena, Romina Bono, Flavia Abruzzese, Sara Cesano, Patrizio Conte, Chiara Airoldi, Pierluigi Neri, Alessandro Carriero.

The original article has been corrected.

